# Resveratrol decreases extracellular traps (ETs) in acute promyelocytic leukemia (NB4) cells

**DOI:** 10.1371/journal.pone.0321221

**Published:** 2025-04-17

**Authors:** Mahshid Vafajoo, Minoo Shahidi, Fahimeh Shahriyary, Mohammad Reza Amirzargar, Ahmad Kooshari

**Affiliations:** Department of Hematology and Blood Banking, School of Allied Medical Sciences, Iran University of Medical Sciences, Tehran, Iran; Tekirdag Namik Kemal University: Tekirdag Namik Kemal Universitesi, TÜRKIYE

## Abstract

**Background:**

Activated neutrophils can create structures known as neutrophil extracellular traps (NETs/ETs) consisting of nuclear components and granules. The ETOsis phenomenon leads to activating the platelets and coagulation factors. Accordingly, coagulation and fibrinolysis can be promoted. Resveratrol (RSV) is a botanical antioxidant with anti-inflammatory and anti-leukemia effects. The present study was conducted to assess the effect of RSV on the occurrence of ETOsis in the NB4 cell line.

**Methods:**

Human acute promyelocytic leukemia cell line (NB4) were stimulated and treated by lipopolysaccharides (LPS) and RSV, respectively. Sytox green and a fluorescent microscope were used to assess the ETOsis in NB4 cells. Furthermore, the expression level of peptidylarginine deiminase 4 (PAD4) gene and the occurrence of ETOsis in NB4 cells were evaluated by real-time PCR and flow cytometry, respectively. Moreover, an enzyme-linked immunosorbent assay (ELISA) kit was utilized to measure tumor necrosis factor-α (TNF-α) cytokine.

**Results:**

Following treatment with RSV, a significant decrease in PAD4 gene expression and TNF-α cytokine concentration in the supernatant of NB4 cell line culture medium was observed. Besides, the amount of ETOsis in the NB4 cells treated with LPS and RSV decreased.

**Conclusion:**

The findings demonstrated that RSV can inhibit the process of ETOsis in NB4 cells. By inhibiting the process of ETOsis, RSV may be able to reduce the bleeding and, consequently, the failure after treatment in acute promyelocytic leukemia (APL) patients.

## Introduction

Neutrophils have a killing function by engulfing and degrading bacteria through antimicrobial cytoplasmic granules [1]. In addition, activated neutrophils have the ability to destroy extracellular pathogens through forming neutrophil extracellular traps (NETs/ETs), consisting of nuclear components and granules. Interleukin 8, phorbol myristate acetate (PMA), and lipopolysaccharides (LPS) are factors that could stimulate neutrophils for NETs formation [2].

During NET formation, peptidylarginine deiminase 4 (PAD4) is activated by increasing the intracellular calcium level. Active PAD4 converts arginine in histones to citrulline after translocation into the nucleus. Finally, the chromatin is decondensed as a result of the lack of the positive charge of histones [3]. Therefore, a particular marker for ETosis could be citrullinated histone. Given the observed relationship between ETosis and thrombosis in diseases such as cancer-associated thrombosis, the risk of thrombosis can be evaluated by the specific markers of ETosis [4]. Moreover, increasing the level of DNA in the blood circulation can be considered one of the molecular mechanisms of blood clotting and thrombosis in cancers [5].

Acute promyelocytic leukemia (APL) is a kind of blood cancer which occurs as the result of the t (15,17) chromosomal translocation. In this disease, some cellular processes, including the division, differentiation, and death of promyelocytes, are disrupted [6]. These patients are involved in thrombotic and bleeding coagulation problems, which are life-threatening [7]. All-*trans* retinoic acid (ATRA) and arsenic trioxide (ATO) drugs are used to treat APL, which induce both apoptosis and cell differentiation. But unfortunately, in some cases, due to patients becoming resistant to these drugs, the disease will recur after a while [8]. ATO has shown a dose-dependent effect on ETOsis and apoptosis processes, with its medium and high concentrations stimulating ETOsis and apoptosis in APL cells, respectively [9]. Moreover, ATRA can induce and increase the ETOsis process in promyelocytic cells by increasing cytokines such as TNF-α and IL-6 [6]. Promyelocyte extracellular chromatin induced by ATRA causes excessive use of coagulation factors, thrombin production, and fibrin deposition. Ultimately, these factors cause disruption of clot lysis and endothelial cell damage [7]. In addition, ETs results in the activation of platelets, whereby the secretion of active platelet factors, the stimulation of endothelial retraction, and the creation of gaps between endothelial cells happen. Consequently, the leakage of red blood cells (RBCs) from the vessels leads to bleeding. Therefore, it seems useful to find a solution to reduce the occurrence of ETOsis and, subsequently, the bleeding burden and more effective treatment in APL patients [10].

Resveratrol (RSV) (3,5,4 -trihydroxy-trans-stilbene) is a small polyphenol, which is found in sources such as red grapes and various berries. It is considered a strong antioxidant that reduces the cytokines and pro-inflammatory mediators such as IL-6 and TNF-α in immune cells by destroying reactive oxygen species (ROS) and inhibiting nuclear factor-kappa B (NF-κB) [11,12]. As studies revealed, RSV and ATO have a synergistic pro-apoptotic effect and anti-leukemia activity on NB4 cells [13]. Moreover, RSV induces apoptosis and differentiation of APL cells. The findings state that the simultaneous use of RSV and ATRA increases differentiation in NB4 cells, so further investigation of RSV as a therapeutic agent for APL can be beneficial [14].

One of the main problems in patients with APL is disseminated intravascular coagulation (DIC), a tendency to thrombosis and bleeding [15]. Additionally, the use of ATRA, by increasing the occurrence of ETOsis in APL cells, can aggravate thrombosis and bleeding in these patients [7]. Given the anti-inflammatory effect of RSV, it seems interesting to investigate its effect on the process of ETOsis in these patients. Due to these reasons, this study assessed whether RSV inhibits ETOsis in acute promyelocytic leukemia (NB4) cells stimulated by LPS or not.

## Method and material

This research was conducted on the NB4 cell line gifted by Dr. Majid Safa at the Hematology Research Center of Iran University of Medical Sciences.

### Cell culture and drug treatment

Human acute promyelocytic leukemia cell line (NB4) was cultured in RPMI-1640 with L-Glutamine (Gibco) medium containing 10% fetal bovine serum (FBS) (Cegrogen Biotech) and 1% penicillin-streptomycin (Penicillin 100 U/ml-Streptomycin 100 µg/ml) (Gibco) at a 5% CO2 humidified atmosphere and 37°C. The cells in the log phase were subjected to experiments.

Seeded NB4 cells in 6-well plates at 10^5^ cells/well were stimulated with LPS (500 ng/mL, Sigma Aldrich). Then, they were treated with different concentrations (0, 5, 10 μM) of RSV (Sigma Aldrich) for 2:30 h. The drug concentrations were selected regarding the obtained results in the MTT assay. In the control well, cells were only incubated with RPMI-1640 supplemented medium.

### MTT assay

After harvesting NB4 cells treated with certain concentrations of RSV (Sigma Aldrich) and control cells (media alone), they were incubated for 3 hours at 37°C. Approximately 7 ×  10 ^4^ of these cells were cultured in 94-well plates. Then, thiazolyl blue tetrazolium bromide (MTT) solution (Sigma Aldrich) with a concentration of 0.5 mg/ml was added to the cells. After 3 hours, the cells were centrifuged at 1000 g for 10 minutes. In order to dissolve the formazan, 100 μl of dimethyl sulfoxide (DMSO) solution was added to the wells and mixed on a shaker for 15 minutes. Then, the optical density (OD) was read by an ELISA reader at wavelengths 570–630. It should be noted that the OD of the control sample is considered 100% viability. Moreover, all tests were performed in triplicate.

### Quantitation of ETs formation by flow cytometry (SYTOX green)

NB4 cells (15 × 10^4^) were resuspended in RPMI-1640 supplemented with 10% FBS, seeded in 24-well plates, and subjected to stimulation [LPS (500 ng/mL)] along with different concentrations (0, 5, 10 μM) of RSV. They were incubated for 2:30 hours at 37^◦^C in a CO_2_ incubator. After the drug treatment step, SYTOX green (10 nM, Biolegend, USA) was added to each well. One well is unstained. The chromatin released by NB4 cells is bindable by SYTOX green, a cell impermeable dye that binds to extracellular DNA. The ETs process was quantified using SYTOX green by FACS Calibur flow cytometry (Becton Dickinson). Then, the results were analyzed with FlowJo_V10 software (BD Biosciences, USA).

### Quantitation of ETs formation by fluorescence microscopy

After the drug treatment step, the ETs were stained with SYTOX green (1 µ M, Biolegend, USA), and Images were taken on a fluorescent microscope (NIKON ECLIPSE Ts2R), and the results were analyzed by Image J (NIH, USA).

### PCR assay

In order to assess the changes in PAD4 gene expression in NB4 cells stimulated with LPS and treated with the RSV drug, the qRT-PCR technique was performed. After the treatment step, the cell sediment was isolated, and total RNA was extracted using RNX-Plus Solution (EX6101, Sinaclon). Then, cDNA was synthesized according to the manufacturer’s instructions for the cDNA Synthesis Kit (Ana cell). To perform the qRT-PCR, SYBR Green RT-PCR Master Mix (Ampliqon), forward primer, reverse primer, cDNA sample, and double-distilled water were used. After that, the samples were subjected to 40 cycles as follows: each cycle including 15 seconds at 95°C and 60 seconds at 60°C. Each reaction was repeated three times. It should be noted that GAPDH gene was used as an internal control. Finally, the results were calculated using the ∆∆Ct method.

PAD4 primer was designed using the website http://www.ncbi.nlm.nih.gov/blast/ and Oligo 7 software. The primers sequences were listed in Table 1.

**Table 1:** Sequences of primers used for quantitative PCR (QPCR).

Gene	Forward	Reverse
PAD-4	5′- GTGACCCTGACGATGAAAGTG-3′	5′-CGGTGAGGTAGAGTAGAGCTT-3′
GAPDH	5′-AAGGTCGGAGTCAACGGATTTG-3′	5′-GCCATGGGTGGAATCATATTGG-3′

### Enzyme-linked immunosorbent assay (ELISA)

The cell supernatant was separated through centrifuging at 2500 RPM for 10 minutes at 4°C. Finally, TNFα was quantified in cells supernatant according to the ELISA Kit (IBL, Hamburg, Germany) manufacturer’s instructions.

### Statistical analysis

The data was analyzed by GraphPad prism 8.2.1 software (GraphPad Software, Inc., San Diego, CA, USA). The mean ±  SD was calculated to describe quantitative variables. In order to compare quantitative variables between two and more than two independent groups, student’s *t* test or one-way ANOVA were used, respectively. *p* value less than 0.05 was regarded as significant.

## Results

### The cell viability of NB4 cells using the MTT assay

In the presence of different concentrations of RSV, viability was detected in NB4 cells. The MTT results considered a dose of 5 µ M as the best-selected dose.

### Flow cytometry for ETs quantitation

**Fig 1:**
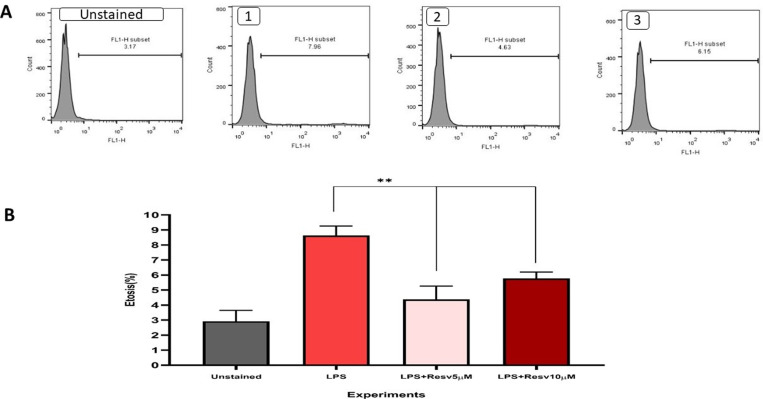
A. Representative data of NB4 cells (treated with LPS and RSV) ETOsis percentage by Flow cytometry method. NB4 cells are stimulated with LPS (1). NB4 cells are treated with 5 µ M RSV (2). NB4 cells are treated with 10 µ M RSV. **p < 0.01. In comparison to the condition in which the cells were treated with the stimulant (LPS) alone, 5 and 10 μM resveratrol significantly reduced ETosis. B. Results of flowcytometry analysis.

The results of flow-cytometric analysis showed significant changes between treated groups with RSV 5 and 10 µ M and LPS-stimulated NB4 cells. Both concentrations of RSV significantly decreased the rate of ETosis. However, RSV 5 µ M demonstrated a higher effect compared with 10 µ M (Fig 1; A1 and A2 p = 0.002, Fig 1; A1 and A3 p = 0.002).

### Fluorescence microscopy for ETOsis visualization

**Fig 2:**
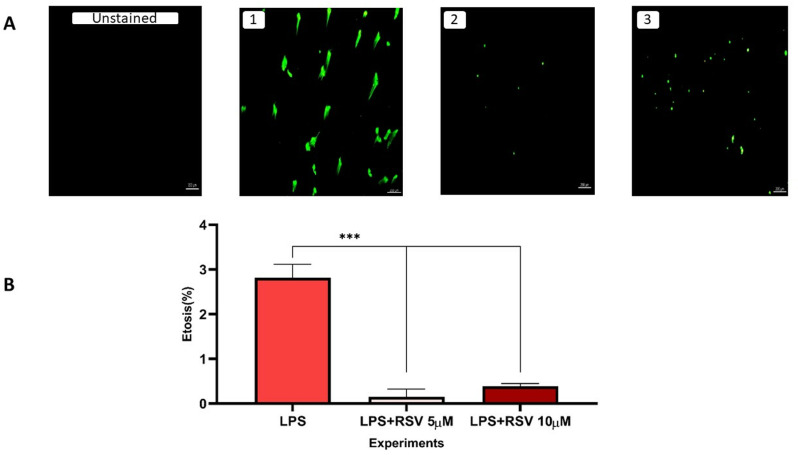
Detection of ETs using SYTOX green. A: LPS stimulated NB4 cells with the SYTOX green concentrations (1). NB4 cells treated with RSV (5 µ M) (2). NB4 cells treated with RSV (10 µ M) (3). All images are 10x,***p < 0.001. Resveratrol (RSV) B: Results of microscopy analysis. In cells treated with resveratrol at both concentrations of 5 and 10 μM, ETOsis incidences were lower than in cells treated with LPS alone.

**Fig 3:**
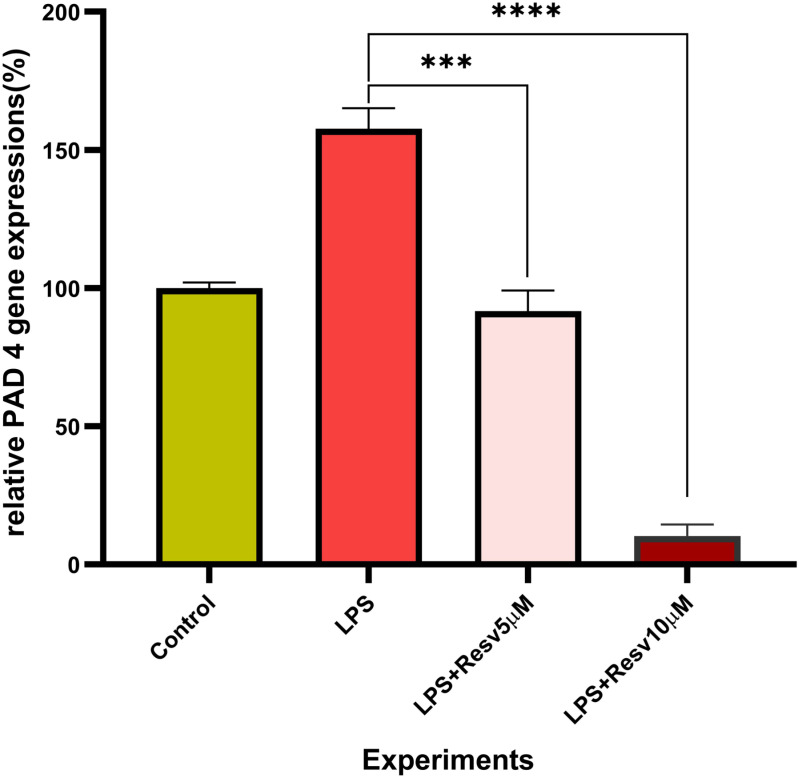
PCR results demonstrated PAD4 expressions in the NB4 cells. ***p < 0.001, ****p < 0.0001. PAD4 expression was increased in NB4 cells treated with LPS stimulus, whereas it was decreased when cells were treated with 5 or 10 μM resveratrol.

In Fig 2, we showed the results of NB4 cells treated with SYTOX green, which was detected by a fluorescent microscope (Fig 2A). The results were analyzed using Image J after counting cells in ten fields for each condition (Fig 2B). In agreement with flow cytometry analysis, results demonstrated a significant decrease in ETOsis formation when compared with RSV-treated NB4 cells (Fig 2; 1A and 2A p < 0.001, Fig 2; 1A and 3A p < 0.001).

### Real-time PCR analysis of PAD4 gene expression

The mRNA levels of PAD4 were assessed as one of the key factors to determine the status of ETOsis. As shown in Fig 3, PAD4 expression level in NB4 cells has significantly decreased in cells treated with both concentrations of RSV 5 M (p < 0.001) and 10 M (p < 0.001) compared to the control group.

### The results of quantification of TNF-α (ELISA)

**Fig 4:**
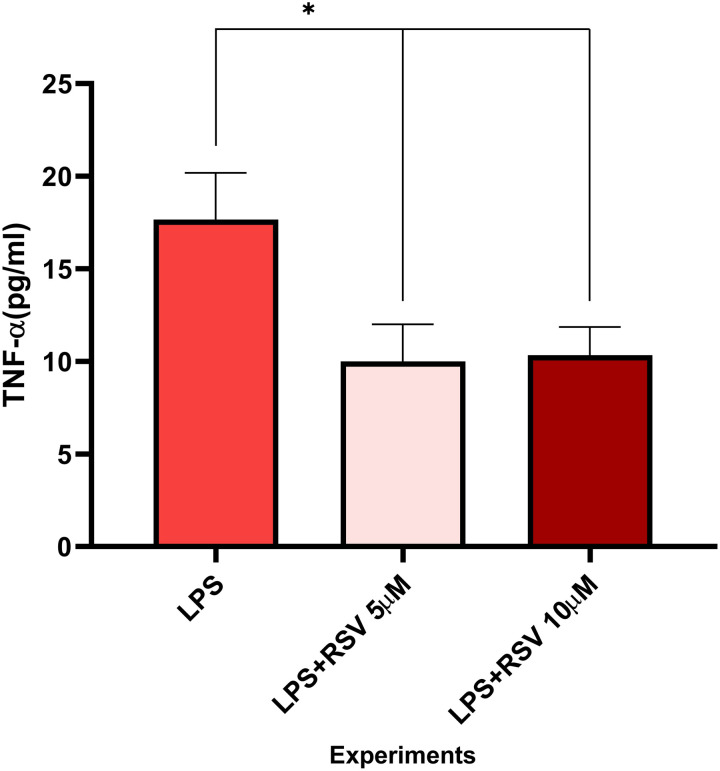
Comparison of TNF- **α** **in RSV-treated groups and the control.** The level of TNFα in RSV-treated groups is significantly lower than control (LPS). Individual data are presented as mean ±  SD (t test), **p < 0.01.

The measurement of TNF-α by ELISA indicated a significant decline in the level of cytokine in the RSV treatment groups with concentrations 5 µ M (p =  0.014) and 10 µ M (p =  0.012) rather than control group (Fig 4, Table 2).

**Table 2:** The results of the occurrence of ETOsis from the tests performed.

The results of:	LPS(Control)	LPS+RESV 5 µ M	LPS+RESV 10 µ M
**Flow cytometry**	The incidence of ETOsis increased	Compared to the control state, it had the greatest decrease	It was reduced compared to the control state
**Fluorescence microscopy**	The incidence of ETOsis increased	It was reduced compared to the control state	It was reduced compared to the control state
**Real-time PCR**	The expression level of PAD4 was increased.	The expression level of PAD4 was reduced compared to the control state	The expression level of PAD4 was the most decreased compared to the control state
**ELISA**	The level of TNFα was increased	The level of TNFα was decreased compared to the control state	The level of TNFα was decreased compared to the control state

The incidence of ETOsis increased in the control state when the cells were treated with LPS alone in all assays, while ETOsis decreased when resveratrol was added to the medium at both doses. Additionally, in cells treated with LPS stimulant only, expression of the PAD4 gene, a key marker of ETOsis, increased, while in cells treated with resveratrol, it decreased. Compared to the control condition, TNF-α decreased in cells treated with resveratrol. Thus, resveratrol inhibited ETOsis in NB4 cells.

## Discussion

The present study investigated the effect of RSV on the occurrence of ETOsis in the NB4 cell line. The findings of flow cytometry and microscopy demonstrated that the RSV reduces ETosis in NB4 cells. Moreover, a decrease in ETOsis was observed in NB4 cells treated with LPS stimulus and RSV. For the first time, in 2016, it was demonstrated that APL cells were affected by ETOsis through the release of extracellular DNA traps (ET). It was also found that ATRA accelerated ET formation by increasing the levels of TNF-α, interleukin-6 and autophagosome formation [6]. Researchers suggest that autophagy in APL cells is induced by ATO through rapamycin-dependent autophagy and reactive oxygen species. Leukaemia-initiating cells (LICs) are reduced by increased ETosis caused by ATO. Through ETosis, ATO exerts anti-leukaemic effects and targets LICs as the key component of the treatment and relapse prevention protocol. Therefore, increasing ETosis induced by ATO seems like a potential strategy for eradicating LICs[9]. This theory, however, has been contradicted by other studies; observations have demonstrated that ATRA causes ETosis, which results in extracellular chromatin that produces excessive amounts of thrombin and fibrin, increases plasmin, and damages endothelium. Extracellular promyelocytic chromatin exacerbates the coagulation and fibrinolysis of acute promyelocytic leukemia and may cause induction failure in high-risk patients with APL [7]. A study published in 2022 demonstrated that ATRA and ATO drugs induce and increase ETOsis in mature neutrophils. Moreover, platelet-derived factor 4 stimulates ETOsis, causing ETs to stimulate platelets and secrete platelet derivatives, resulting in a positive feedback loop. ETs lead to endothelial contraction and gap formation in APL, which result in RBC leakage and increased bleeding. Therefore, an ETOsis inhibitor may reduce bleeding in APL and protect endothelial cells from damage [10].

RSV is a botanical ingredient with anti-inflammatory, anti-tumor, and anti-leukemia effects [16]. Considering the previous studies, RSV can inhibit cell proliferation in NB4 cells through apoptosis. Certainly, further studies on this drug as an effective therapeutic agent in APL patients is useful [13,14]. Our results demonstrated the occurrence of ETOsis in the NB4 cells in the presence and absence of RSV. In order to quantitatively investigate the occurrence of ETOsis, the PAD4 gene expression change was measured using the real-time PCR technique. The results of our study revealed that the expression of the PAD4 gene decreased significantly after treatment of cells with RSV compared to LPS stimulation alone. The highest decrease was observed in 10 µ M concentration. Therefore, compared to the findings of flow cytometry and microscopy, it is quite obvious that the RSV reduces the occurrence of ETosis in NB4 cell line. However, to find the most suitable concentration of RSV, more investigation is needed.

There is still a lack of understanding of how resveratrol reduces ETOsis. Future research may help to resolve this question. Resveratrol, this phenolic molecule, has been shown to inhibit the reactive oxygen species (ROS) cycle (COX) in previous studies. Additionally, it activates anti-inflammatory pathways, such as SIRT1, which reduces the production of inflammatory factors such as TNF-α. And activates many anti-inflammatory pathways, including SIRT1, to reduce the production of inflammatory factors like TNF, IL-1, IL-6, MMP-1 and COX-2 by inhibiting nuclear factor-kappa-B (NF-KB) [11]. Finally, considering that the ETOsis process requires ROS from the NADPH oxidase pathway and inflammatory cytokines such as TNF-α, IL-6, resveratrol may inhibit the ETOsis process by reducing these products.

In a previous study, it was shown that in NB4 cells treated with ATRA drug, the concentration of cytokines IL-6 and TNF-α increased in a time-dependent manner. The increase of these cytokines causes the stimulation of NB4 cells and the release of ETs (Extracellular Traps) by these cells [6]. Moreover, the RSV drug in combination with ATRA induces both apoptosis and differentiation in NB4 cells [14] and it shows a synergistic anti-leukemia effect when used in conjunction with ATO [13]. Furthermore, we observed that resveratrol significantly decreased VWF, t-PA-1, and IL-8 levels in our previous study of the effects of resveratrol on human umbilical vein endothelial cell expression and secretion of coagulation, fibrinolytic, and inflammatory markers. Additionally, resveratrol reduced VWF and t-PA-1 mRNA expression as well as factor VIII activity. In cell culture (in vitro), resveratrol demonstrated anti-inflammatory, anticoagulant, and anti-fibrinolytic effects [17].

In the present study, the amount of TNF-α cytokine in the supernatant of the NB4 cell line culture medium stimulated with LPS increased. The amount of this cytokine in the supernatant of NB4 cell line culture medium treated with LPS in the presence of RSV at both 5 and 10 μM concentrations has decreased compared to the control state (treatment with stimulant alone). In another study on the RSV, it has been suggested that the simultaneous use of RSV and ATO can reduce the hepatotoxicity resulting from ATO [18]. In addition, recent findings on animal models revealed that the RSV with its strong antioxidant potential prevents the accumulation of arsenic in the liver and kidneys in rats exposed to ATO [19]. Furthermore, APL cells in patients treated with ATO undergo autophagy-induced ETOsis [9]. Recent studies have also shown that there is a significant positive correlation between plasma ATO concentration and cfDNA concentration in elderly APL patients treated with ATO and found that cfDNA in these patients is caused by the ETOsis process [20]. Moreover, RSV can inhibit PI3K/AKT pathway activity in NB4 and HL-60 cells by upregulating the expression of phosphatase and tensin homologue (PTEN), whereby the proliferation of leukemic cells is suppressed, and apoptosis is induced in these cells. Therefore, it has been claimed that RSV has an anti-leukemia effect [21]. Since RSV has recently been suggested to be an agent inducing apoptosis and modulating autophagy in APL cells, it can be considered as an efficient chemotherapy agent [22].

Although ATRA and ATO are used to treat patients with APL, the development of thrombosis and embolism is regarded as the main problem after treatment [10]. For the first time, the current study has investigated the effect of RSV on ETOsis of APL (NB4) cells, which is one of the causes of thrombosis. It could be a turning point to find a less complicated treatment in the future.

The limitations of this research should be considered. The first limitation is the use of NB4 cells instead of bone marrow (BM) cells and plasma from APL patients, which would yield more accurate results. However, due to the difficulty of preparing BM samples and maintaining and cultivating them, NB4 cell line was used in this study. Moreover, if ATRA and ATO are used in combination with RSV, the results will be more reliable for use in clinical settings where patients are treated with these drugs.

## Conclusion

In conclusion, RSV causes a decrease in PAD4 gene expression and TNF-α cytokine concentration in the supernatant of NB4 cell line culture medium. Furthermore, the amount of ETOsis in the NB4 cell line treated with LPS and in the presence of RSV, decreased. Therefore, the RSV, in addition to being an effective therapeutic agent in APL patients, may be able to reduce burden of bleeding by inhibiting the process of ETOsis and subsequently reduce failure after treatment in APL patients. It is suggested to use RSV in combination with ATRA in future studies and investigate the effect of RSV on the ETOsis process in NB4 cell line treated with ATRA, so that a more efficient treatment for APL patients can be approached. Additionally, it is suggested that animal samples be used in future studies to investigate the effect of RSV on the ETOsis process in APL cells in order to obtain clinically relevant results.

## Supporting information

S1 Data Supplementary information data. Real-time PCR data, statistical analysis, and fluorescent microscopy images. (RAR)
